# Acid–base and electrolyte abnormalities in heart failure: pathophysiology and implications

**DOI:** 10.1007/s10741-015-9482-y

**Published:** 2015-03-28

**Authors:** Caterina Urso, Salvatore Brucculeri, Gregorio Caimi

**Affiliations:** Dipartimento Biomedico di Medicina Interna e Specialistica, Universitá di Palermo, 90127 Palermo, Italy

**Keywords:** Acid–base disturbances, Congestive heart failure, Electrolyte abnormalities

## Abstract

Electrolyte and acid–base abnormalities are a frequent and potentially dangerous complication in subjects with congestive heart failure. This may be due either to the pathophysiological alterations present in the heart failure state leading to neurohumoral activation (stimulation of the renin–angiotensin–aldosterone system, sympathoadrenergic stimulation), or to the adverse events of therapy with diuretics, cardiac glycosides, and ACE inhibitors. Subjects with heart failure may show hyponatremia, magnesium, and potassium deficiencies; the latter two play a pivotal role in the development of cardiac arrhythmias. The early identification of these alterations and the knowledge of the pathophysiological mechanisms are very useful for the management of these patients.

## Introduction

Heart failure is a major cause of cardiovascular mortality and morbidity, resulting in more than one million hospitalizations per year in the USA, and it is the most common hospital discharge diagnosis among subjects older than 65 years [1].

Subjects with congestive heart failure (CHF) usually show acid–base and electrolyte disorders, due both to the activation of several neurohumoral mechanisms and to drugs used in this condition, such as diuretics [2]. These abnormalities reflect the severity of CHF and contribute to the functional impairment and to the poor long-term prognosis [3].

The common electrolyte abnormalities are hyponatremia, hypokalemia, and hypomagnesemia. The acid–base disturbances generally observed are metabolic alkalosis pure or combined with respiratory alkalosis [4]. Several mechanisms interact to produce these alterations. The decrease in cardiac output leads directly to a reduction in renal blood flow, with impairment of renal excretion of water and electrolytes, and it causes the activation of several neurohormonal responses which affect both cardiovascular homeostasis and electrolyte balance. The therapy of CHF subjects includes the discovery and management of these electrolyte abnormalities that have a role in the development of ventricular arrhythmias [5].

## Hyponatremia

Hyponatremia is the most common electrolyte abnormality observed in hospitalized subjects; it is defined as a serum sodium concentration lower than 136 mmol/L [6]. Mild-to-moderate hyponatremia is generally present in 10 % of HF subjects [7]; however, this frequency seems to be higher in different reports. For example, in the OPTIME-CHF trial, 27 % of subjects show serum sodium concentrations ranging between 132 and 135 mEq/L [8], while in the ESCAPE trial, 18 % of the subjects had persistent hyponatremia throughout their hospitalization, defined as serum sodium below 134 mEq/L [3].

Maintenance of total body salt and fluid within normal range is under the control of the atrial-renal reflexes, the RAAS, and the sympathetic nervous system (SNS) [9].

**Fig. 1:**
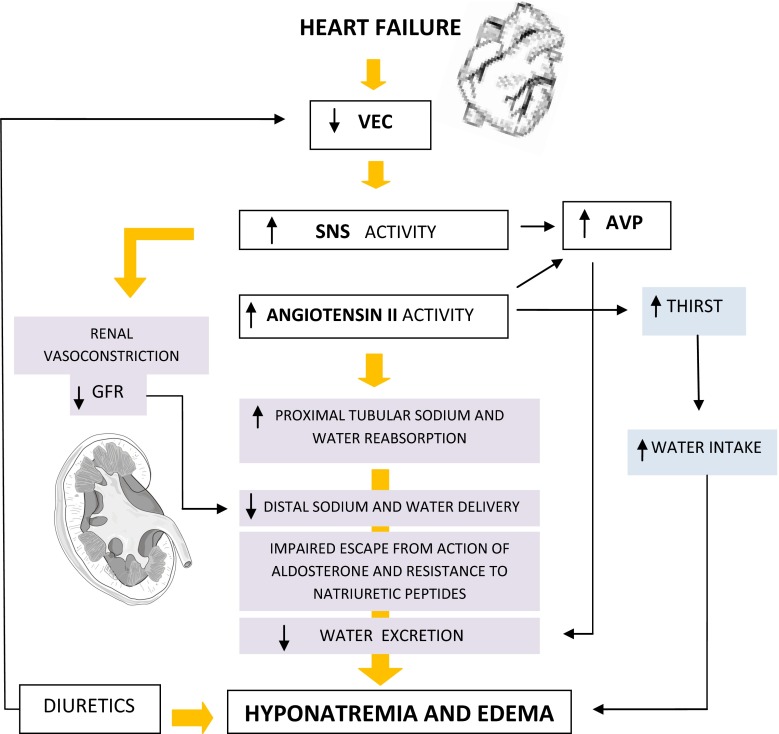
Hyponatremia in heart failure. *VEC* extracellular fluid volume, *SNS* sympathetic nervous system, *AVP* arginine vasopressin, *GFR* glomerular filtration rate

In a normal heart, any increase in atrial pressure suppresses the release of the antidiuretic hormone, decreases the tone of the SNS in the kidneys, and enhances the release of the atrial natriuretic peptide [10]. The latter promotes sodium and water excretion at the distal nephron, improves GFR, causes vasodilatation, and decreases the release of the antidiuretic hormone. In HF, these actions are blunted, and therefore sodium and water retention occurs despite elevated atrial pressures [11]. The decrease in cardiac output and in effective circulating volume leads to activation of baroreceptors, which in turn activate the SNS, the RAAS, and the release of arginine vasopressin. The final effect is an enhanced retention of sodium and water [12, 13] (Fig. 1).

The SNS activation helps to maintain circulatory homeostasis and perfusion to vital organs by increasing inotropy and chronotropy of the failing myocardium and by modulating vascular reaction. In the long term, these mechanisms as well as the RAAS activation are maladaptive and responsible for disease progression [14–16].

Furthermore, it is well known that heart and kidneys are closely interrelated and that disorder of either of the two organs may induce dysfunction in the other organ in a spiral fashion leading to cardiorenal syndrome (CRS). These two organs act in tandem to regulate blood pressure, vascular tone, diuresis, natriuresis, intravascular volume homeostasis, and peripheral tissue perfusion. Changes in the RAAS, SNS, and inflammation are the cardiorenal connectors to develop CRS [17].

As cardiac output drops, renal blood flow and GFR follow suit, impairing the kidney’s ability to excrete dilute urine. Dilutional impairment occurs due to disturbance of one or more of the following mechanisms: GFR, separation of salt and water at the thick limb of Henle’s loop, and ADH action in the collecting duct. The volume of tubule fluid that is delivered to the distal nephron determines in large measure the volume of dilute urine that can be excreted. Thus, if glomerular filtration is decreased or proximal tubule reabsorption is greatly enhanced, the resulting diminution in the amount of fluid delivered to the distal tubule itself limits the rate of renal water excretion [18, 19].

Angiotensin II promotes the retention of sodium and water by the stimulation of the release of aldosterone, increasing the efferent arteriolar tone, hence promoting sodium and water absorption by the resulting rise in the filtration fraction, as well as by a direct effect on proximal tubule [20], by stimulating the thirst center, and by causing the release of arginine vasopressin [7].

Both adrenergic stimulation and angiotensin II activate receptors on the proximal tubule epithelium, leading to increased sodium reabsorption and decreased sodium and water delivery to the renal collecting duct, thus exacerbating the sodium retaining effect of aldosterone and reducing the diuretic effects of natriuretic peptides [12]. Furthermore, angiotensin II activates NADPH oxidase, which results in the formation of reactive oxygen species. The increased oxidative stress enhances negative inotropic effects and induces cardiac remodeling [21]. Therefore, a vicious cycle sets in promoting structural and functional damage to both kidneys and heart. Aldosterone, in turn, causes continuous renal sodium reabsorption and increases the myocardial fibrosis of the failing heart [22].

Hyponatremic subjects with advanced HF often have inappropriately elevated plasma AVP levels that in turn, lead to enhanced renal water retention by increasing the number of aquaporin water channels in the collecting duct of the kidney [23].

The actions of AVP are mediated by three AVP receptor subtypes (V1aR, V1bR, and V2R). The V1aR is located in vascular smooth muscle cells and cardiomyocytes mediating vasoconstriction and hypertrophy, platelets aggregation, and glycogen metabolism in hepatocytes. The V1bR located in the anterior pituitary play a crucial role in regulating hypothalamic–pituitary–adrenal axis activity by stimulating the release of corticotrophin and ACTH. The V2R located in the collecting tubules of the kidney are notable in the pathophysiology of HF and mediate the antidiuretic effect of AVP. Binding of AVP to V2R activates the adenylate cyclase signaling pathway, leading to phosphorylation of the preformed water channel aquaporin-2 and their subsequent insertion into the apical membranes of the collecting ducts. Furthermore, the urinary excretion of aquaporin-2 is increased in heart failure subjects with elevated AVP [24]. Notably, the elevated plasma AVP levels are not adequately reduced even with acute water loading in hyponatremic HF subjects [23].

The AVP cannot be reliably investigated by the current laboratory methods; however, copeptin, the C-terminal segment of the AVP precursor peptide, is secreted in an equimolar ratio to AVP and is a sensitive and stable surrogate marker for its release. Copeptin is also a promising indicator in the differential diagnosis of hyponatremia [25].

Hyponatremia may be a marker of neurohormonal activation that reflects the severity of heart failure [26], but it may also result from the HF therapy [7, 27].

Diuretics are one of the most common causes of drugs induced hyponatremia; Although thiazide diuretics are most often implicated [28], also non-thiazide agents, such as furosemide, spironolactone, and indapamide, have been associated with hyponatremia [29].

It should also be mentioned that the hydrochlorothiazide and amiloride combination increases the risk of hyponatremia. This increment is probably due to the direct effect of amiloride on the collecting tubule increasing sodium loss. Moreover, amiloride spares potassium and, therefore, worsens thiazide-induced hyponatremia as a consequence of potassium retention by exchanging it for sodium in the distal tubule [30].

Several clinical studies have shown that hyponatremia is associated with adverse prognosis and reduced survival in HF [31]. Serum sodium concentration on admission or discharge is a predictor of in-hospital short-term and long-term mortality in subjects hospitalized for HF [32, 33]. Moreover, hyponatremia is associated with an increased rate of re-hospitalization and major complications [32], as well as a longer hospital stay in hospitalized HF subjects [34, 35]. In a study of 355 subjects admitted for HF, a serum sodium concentration <130 mEq/L was associated with a higher in-hospital death rate [36]. In the OPTIME-CHF study, subjects with serum sodium <135 mEq/L had longer lengths of hospital stay and a doubling of in-hospital as well as 60-day mortality [8]. Finally, serum sodium levels also predict mortality in outpatients with chronic heart failure [33, 37].

The treatment of significant hyponatremia in heart failure is not easy. The conventional treatments such as fluid restriction, infusion of hypertonic saline, and conventional diuretic therapies are not usually effective. Vasopressin receptor antagonists have been shown to enhance aquaresis and correct hyponatremia. However, long-term beneficial effects of such treatments in chronic heart failure have not been validated [38].

## Hypokalemia

Hypokalemia is commonly observed in CHF subjects, and it is a strong independent predictor of mortality [39].

Hypokalemia has not been well defined in HF, and even in the literature, its range varies from 3.5 to 4.0 mEq/L (mmol/L) [40]. Hypokalemia is generally more evident in subjects with advanced CHF receiving pronounced diuretic therapy and with the greatest activation of the renin–angiotensin system [41].

Low levels of serum K^+^ may be a marker of increased neurohormonal activity and disease progression [42]; furthermore, serum K^+^ is negatively correlated with plasma renin activity and plasma noradrenaline [43].

Catecholamines cause hypokalemia and increase the arrhythmic risk; adrenaline, in fact, stimulates the sodium–potassium-ATPase pump via β_2_-receptors and shifts potassium intracellularly. It seems likely that the observed mortality benefit with beta-blockade in HF is partly ascribable to the prevention of hypokalemic arrhythmias [44].

It is known that potassium depletion is a risk factor for increased frequency of ventricular arrhythmias; moreover, hypokalemia can potentiate the arrhythmias associated with CHF therapy (e.g., digitalis) and diminish the efficacy of anti-arrhythmic drugs by altering the electrophysiologic properties of the myocardium and negating some of the antiarrhythmic activity of these agents. The frequency of ventricular ectopic beats and the frequency of sudden death correlate with both serum and whole body levels of potassium [45].

A total of 50 % of deaths from HF are sudden, presumably due to arrhythmias. In victims of sudden cardiac death (SCD), the level of myocardial K^+^ is often lower than in controls, and survivors may show hypokalemia apparently caused by a shift of potassium from the intravascular compartment [46]. It is unclear whether hypokalemia precedes and causes the episode or occurs as a result of resuscitation; however, it was found a correlation between the decreased serum K^+^ (<4.4 mEq/L) and SCD [47].

In HF, all-cause and cardiac mortality rates are higher in subjects taking non-K^+^-sparing diuretics; furthermore, the incidence of arrhythmic death is significantly and independently correlated with the use of non-K^+^-sparing diuretics [48].

In HF subjects, there is evidence that the serum potassium level should be maintained above 4.5 mEq/L to minimize the risk of SCD [40, 47, 49], while Leier et al. [50] advise maintaining the level in the range of 4.5 to 5.0 mEq/L. A mild hypokalemia may be corrected by the use of aldosterone receptor antagonists such as spironolactone or eplerenone, while a more severe hypokalemia should preferably be corrected using K^+^ supplement [40]. However, potassium replacement should be routinely considered in patients with CHF, even if the initial potassium determination appears to be normal [51].

Potassium depletion causes diastolic dysfunction [52], while high potassium protects against hypertensive endothelial dysfunction [53, 54].

Potassium mediates vasodilation via strong inwardly rectifying potassium channels and the sodium–potassium-ATPase pump of vascular smooth muscle cells, and this may be useful when NO bioavailability is low; potassium also reduces angiotensin II-induced vasoconstriction [55].

**Fig. 2:**
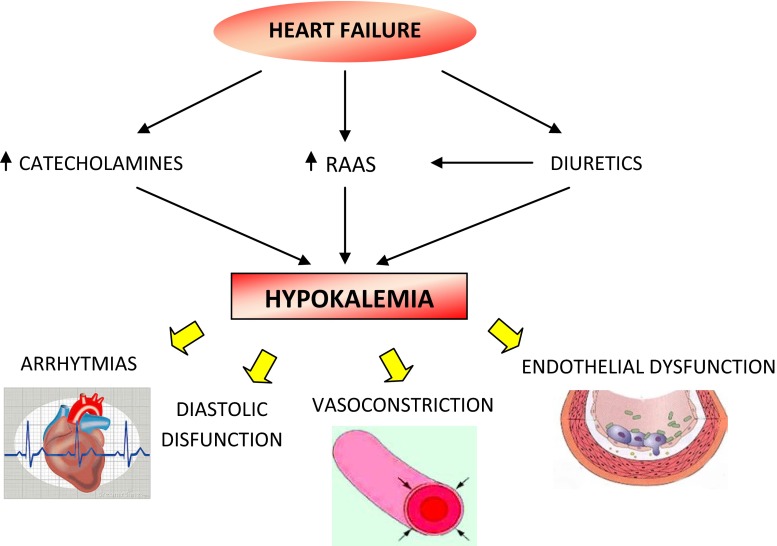
Hypokalemia in heart failure. *RAAS* renin–angiotensin–aldosterone system

In vitro, high extracellular potassium concentration impairs platelet aggregation; moreover, in animal models, increasing plasma potassium reduces the rate of thrombosis on endothelial lesions. Potassium ameliorates oxidative stress by reducing free-radical formation, diminishing vascular smooth muscle cell proliferation, and reducing monocyte adherence to vessels [56]. Furthermore, potassium appears to have an antihypertensive effect mediated by increased natriuresis, vasodilation, heightened baroreflex sensitivity, and reduced cardiac sensitivity to catecholamines and angiotensin II [57]. Potassium also seems to retard the progression of atherosclerosis [56] (Fig. 2).

While diuretics and adrenergic stimulation may cause hypokalemia, neurohormonal blockade using ACE inhibitors, angiotensin receptor blockers, beta-blockers, and aldosterone antagonists may cause hyperkalemia and so the serum K^+^ level should be frequently checked in these subjects [58].

As result of different studies, the myocardiocytes would present mechanisms of adaptation to chronic hypo- or hyper-kalemia. Studies in animal models showed that cardiac muscle was protected against loss of potassium during chronic potassium depletion by an adaptive increase in the density of sodium pump. An increase in the activity and quantity of (Na^+^–K^+^) ATPase in the myocardial tissue would protect it against K^+^ loss. It is not yet known whether these results can be extrapolated to human beings [59–61].

Bartter’s syndrome offers a opportunity to study clinical effects of chronic electrolyte disturbances. Although extracellular concentrations of potassium were usually very low in these subjects, the electrocardiographic changes were slight and arrhythmias were not common. This pattern may reflect an adaptation of the myocardium to hypokalemia [62].

In a study, intracellular K^+^ concentration and ATPase activity of myocardiocytes were measured in early stage of burn injury. The latter accelerates K^+^ efflux current, but inhibits K^+^ influx current; the reduction in Na^+^–K^+^-ATPase activity may be one reason for decrease in intracellular K^+^ concentration after injury [63].

## Hypomagnesemia

Magnesium plays a role in many enzymatic processes, and it is an important component in the mitochondrial structure and function; it modulates cellular potassium permeability and affects calcium uptake and its distribution [64, 65]. Hypomagnesemia (serum magnesium <1.5 mg/dL) is not infrequently observed in CHF subjects, but its pathophysiology remains less studied when compared to other electrolyte alterations. However, there is evidence that early detection and correction of magnesium abnormalities could remedy potentially dangerous arrhythmogenic effects. There is also confirmation that the effective correction of magnesium disturbances is favorable in CHF subjects [66].

Hypomagnesemia occurs either as an isolated disturbance or in association with other acid–base and electrolyte abnormalities; several interrelated mechanisms are implicated in its pathogenesis [67]. As magnesium and potassium are mainly intracellular ions, measurements in serum or plasma are of limited value to assess magnesium status. There was no correlation between the intracellular electrolyte content and the electrolyte levels in plasma, either for mononuclear cells or erythrocytes or for myocardial and skeletal muscle [68].

In the setting of CHF, magnesium depletion stems from reduced dietary intake, altered distribution of the ion, and renal losses. It should also be considered that edematous states, involving the intestinal mucosa, might interfere with the absorption of microelements, such as magnesium. Respiratory alkalosis may produce a decrease in serum magnesium due to a shift of magnesium inside the intracellular compartment. Furthermore, it is well documented that an excessive catecholamine release in decompensated CHF can significantly influence the trans-cellular magnesium shift [69].

Diuretics (loop-acting diuretics in particular) produce most of renal magnesium loss, especially in the volume-expanded setting of CHF and in associated hyperaldosteronism [69].

**Fig. 3:**
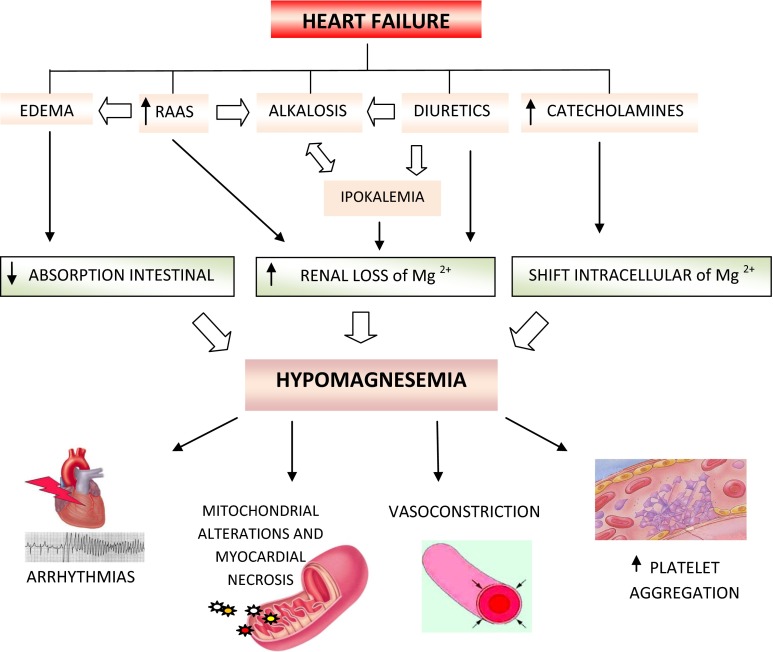
Pathogenesis and effects of hypomagnesemia in CHF. *RAAS* renin–angiotensin–aldosterone system

It has been demonstrated that potassium depletion inhibits the reabsorption of magnesium in the distal convoluted tubule, thus leading to hypermagnesiuria and hypomagnesemia [70]. However, it is well documented that primary disturbances of magnesium balance, particularly magnesium deficit, produce secondary cellular potassium depletion [71] (Fig. 3).

Furthermore, it has been hypothesized that the detection of electrolyte disorders, such as hypokalemia, hyponatremia, hypophosphatemia, hypocalcemia, and especially refractory potassium depletion in CHF subjects, should alert to the possibility of coexisting magnesium depletion [72].

The prevalence of hypomagnesemia in CHF subjects ranges from 7 % of well-compensated ambulatory subjects to 52 % in more advanced CHF subjects who are treated aggressively with diuretics [73].

In animal models, magnesium deficiency leads to the development of mitochondrial alterations with calcium accumulation, cell death, and multifocal myocardial necrosis [74].

Hypomagnesemia seems to have vasoconstrictor properties, secondary to the inhibition of prostaglandin-induced relaxation and to the enhancement of the activity of the vasoconstrictor neurohormones through alterations in calcium uptake. Furthermore, hypomagnesemia can potentially induce hypercoagulability, via enhanced adenosine diphosphate-induced platelet aggregation [75].

The worsening of CHF secondary to very severe hypomagnesemia has been described, and in some cases, magnesium supplementation determines the reversal of heart failure [76].

Moreover, the mortality increase in CHF subjects with hypomagnesemia was believed to be related to the development of ventricular arrhythmias rather than clinical and hemodynamic deterioration. Elevated levels of magnesium decrease the sensitivity of myocardium to the antiarrhythmic action of cardiac glycosides. Digoxin directly limits the renal tubular reabsorption of magnesium, therefore increasing magnesium excretion; a low magnesium concentration increases the action of cardiac glycoside. The antiarrhythmic action of magnesium is mediated by a reduced sensitivity to electrophysiologic changes induced by Ca^2+^. The prognostic significance of serum magnesium concentration in CHF subjects is currently under investigation, although in a retrospective study of subjects with moderate-to-severe CHF, an inverse correlation was noted between mortality and plasma magnesium [77].

## Hypocalcemia and hypophosphatemia

Hypocalcemia (total serum calcium concentration <8.6 mg/dL or ionized calcium concentration <1.1 mmol/L) and hypophosphatemia (serum phosphorus concentration <2.7 mg/dL) are less investigated in HF subjects even though not of minor importance. Despite the pivotal role of calcium ions in contraction of cardiac muscle [78], few cases of hypocalcemia in CHF have been reported and these are often in association with hypomagnesemia [4].

The clinical setting of hypocalcemia includes hypoparathyroidism, end-stage kidney disease, and respiratory alkalosis [79]; in addition, loop diuretics block the reabsorption of calcium in the loop of Henle and may play a role in the pathogenesis of hypocalcemia [80].

It was shown that the correction of calcium disorder could improve CHF [81].

In CHF subjects, hypophosphatemia is generally due to phosphate loss ascribed to respiratory alkalosis, to hypomagnesemia, and to phosphaturic effects of diuretics [82].

Phosphorus depletion has been associated with reversible cardiomyopathy [83].

Some research showed an evident association of increased levels of inorganic phosphate with CHF hospitalization even if the nature of this relationships is not clear [84]; an explanation might be found in features of a myocardiocyte metabolism. It has recently been supposed that inorganic phosphate is both the primary feedback signal for stimulating oxidative phosphorylation and also the most significant product of ATP hydrolysis in limiting the heart capacity to hydrolyze ATP [85].

It is known that inorganic phosphate plays a role in the down-regulation of myocardial contractile force at the onset of ischemia [86]. Furthermore, there is a negative correlation between inorganic phosphates and systolic blood pressure [87].

## Acid–base abnormalities in CHF

In CHF, various acid–base disorders can be discovered due to the renal loss of hydrogen ions and hydrogen ion movements into cells, the reduction in the effective circulating volume, hypoxemia, and renal failure. This justifies the occurrence of metabolic alkalosis, metabolic acidosis, respiratory alkalosis, as well as respiratory acidosis alone or in combination. Several studies have systematically evaluated the prevalence of acid–base disturbances in CHF [88–90]. About 37 % of CHF subjects show at least one acid–base abnormality, most commonly metabolic alkalosis, alone or associated with respiratory alkalosis. Alkalemia is more common in subjects with more advanced CHF (36 % in subjects with class IV compared with 11 % of those with class III) [4]. Other studies confirm the trend toward alkalemia of mixed metabolic and respiratory origin in unselected advanced CHF subjects [88, 90]. In addition, diuretic therapy increases the prevalence of metabolic alkalosis, although the subjects who improve the circulatory status with diuretic therapy may improve their alkalosis [91].

In normal conditions, the kidney preserves normal acid–base balance by bicarbonate reabsorption, principally in the proximal tubule, and bicarbonate generation, predominantly in the distal tubule. Bicarbonate reabsorption is influenced by an effective circulating volume, glomerular filtration rate, and serum potassium level, whereas bicarbonate generation is affected by distal sodium delivery and reabsorption, aldosterone level, arterial pH, and pCO_2_ [92].

In states of volume depletion, the increase in renal avidity for sodium reabsorption results in an acceleration of the sodium–potassium exchange mechanisms, leading to the development of negative potassium balance and favouring the maintenance of metabolic alkalosis [93]. In this case, there is a reduction in bicarbonate back leak from the renal interstitium to the tubular lumen and a consequent increase in the net tubular bicarbonate reabsorption [94].

The renal loss of hydrogen ions in heart failure is related to mineralcorticoid excess and to the increased production of angiotensin II. Mineralcorticoids stimulate the apical sodium channel and basolateral Na^+^–K^+^ ATPase and increased sodium reabsorption promoting secretion through the apical potassium channel. About two-thirds of filtered sodium is reabsorbed with Cl^−^ or in exchange for H^+^ in the proximal tubule. Proximal tubule reabsorption is increased by angiotensin II through the constriction of the efferent glomerular arteriole and through the increase in filtration fraction and in the number of Na^+^–H^+^ exchangers. The final effect is to retain sodium, to deplete potassium, and to produce extracellular alkalosis; furthermore, coexistent potassium depletion increases plasma renin activity and angiotensin II production [95].

**Fig. 4:**
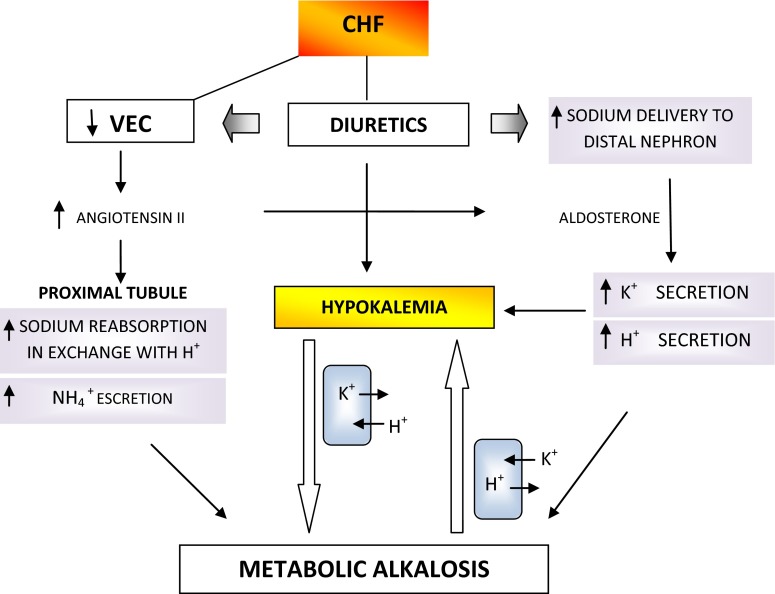
Metabolic alkalosis in CHF. *CHF* congestive heart failure, *VEC* extracellular fluid volume

Diuretics employed in heart failure are frequently responsible for metabolic alkalosis due to several possible mechanisms. Diuretics cause an increase in sodium delivery to the distal nephron and accelerate potassium and proton secretion; furthermore, volume contraction stimulates renin and aldosterone secretion. Potassium depletion with an aldosterone excess is always accompanied by metabolic alkalosis. Hypokalemia promotes alkalosis principally through two mechanisms: first, hydrogen shifts from the extracellular to the intracellular compartments in exchange for potassium, thereby contributing to the alkalemia, and second, hypokalemia produces a stimulation of bicarbonate reabsorption in the proximal tubule and increases the acid excretion [96] (Fig. 4).

Alkalosis seems to be an adverse prognostic factor; in a recent study, the in-hospital mortality rate was higher in the alkalosis group (14.1 %) than in the normal (4.5 %) and acidosis groups (9.3 %) of HF subjects [97].

The treatment of metabolic alkalosis is based on chloride and potassium repletion, enhancement of renal bicarbonate excretion (such as acetazolamide), or, if accompanied by kidney failure, low-bicarbonate dialysis. In CHF, an appropriate management of circulatory failure and the use of aldosterone antagonists in the diuretic regimen are useful for its treatment. In end-stage heart failure, a progressive reduction in plasma renal flow and in GFR leads to renal failure with the reduced capacity of the kidneys to excrete net acid, which can then induce a metabolic acidosis [98].

Light and George [99] evaluated the changes in pulmonary function in 28 subjects hospitalized for CHF, and they showed that initially, subjects had both obstructive and restrictive ventilatory dysfunction. In another study, Niset et al. evaluated the reversibility of the lung dysfunction in 47 patients with severe CHF before and 1 year after heart transplantation. They affirmed that the restrictive ventilatory defect induced by chronic HF was reversible, whereas the exception of the reduction in diffusion lung capacity for carbon monoxide was not improved, which probably reflected irreversible changes in the lung vasculature [100].

Subjects with heart failure and with pulmonary edema can develop respiratory alkalosis in the presence of sustained alveolar hyperventilation that causes hypocapnia. In fact, the stagnation of liquid in the perivascular and peribronchial connective and in the interstitial spaces of the alveolar septa stimulates receptors J and produces a reflex hyperventilation [101].

Effects of acid–base disorders on cardiovascular function are well known. Depression of the cardiac function is observed mainly with respiratory and metabolic acidosis, whereas respiratory alkalosis, mediated by a SNS, interferes on blood pressure and cardiac arrhythmias. The latter are frequently found in metabolic alkalosis associated with hypokalemia [102].

In end-stage heart failure, the most common complication is the pulmonary edema. Positive airway pressure (PAP) therapy represents a potentially beneficial non-pharmacological approach in this clinical condition [103, 104].

PAP diminishes systemic venous return and right ventricular (RV) preload by increasing intrathoracic pressure; it also alters pulmonary total vascular resistance that is the major determinant of RV afterload [105]. In ADHF, PAP therapy increases oxygenation through the recruitment of collapsed alveoli, induces fluid shifts back from alveoli and interstitial space to the pulmonary circulation, reduces respiratory muscle load and the work of breathing, and stabilizes hemodynamics [105].

## Conclusions

CHF subjects develop multiple acid–base and electrolyte abnormalities due to several pathophysiological mechanisms. Their incidence is often correlated with the severity of cardiac dysfunction; furthermore, these imbalances are associated with a poor prognosis. Many of these metabolic derangements are drug-induced; therefore, these subjects need close monitoring. When treating CHF, one must consider how to prevent and to correct electrolyte imbalances.
